# Reduced somatosensory innervation alters the skeletal transcriptome at a single cell level in a mouse model of type 2 diabetes

**DOI:** 10.1038/s41413-025-00436-x

**Published:** 2025-07-04

**Authors:** Masnsen Cherief, Mario Gomez-Salazar, Minjung Kang, Seungyong Lee, Sowmya Ramesh, Qizhi Qin, Mingxin Xu, Soohyun Kim, Mary Archer, Manyu Zhu, Ahmet Hoke, Aaron W. James

**Affiliations:** 1 Department of Pathology, Johns Hopkins University, Baltimore, MD 21205 USA; 2 Department of Neurology and Neuroscience, Johns Hopkins University, Baltimore, MD 21205 USA

**Keywords:** Bone, Type 2 diabetes

## Abstract

Peripheral neuropathy is a common complication in diabetes, affecting around 50% of the diabetic population. Co-occurrence of diabetic peripheral neuropathy (DPN) and diabetic bone disease has led to the hypothesis that DPN influences bone metabolism, although little experimental evidence has yet supported this premise. To investigate, mice were fed a high-fat diet (HFD) followed by phenotyping of skeletal-innervating neurons and bone architectural parameters. Results showed that HFD feeding resulted in a marked decrease in skeletal innervation (69%–41% reduction in Beta-III-Tubulin-stained nerves, 38% reduction in CGRP-stained nerves in long bone periosteum). These changes in skeletal innervation were associated with significant alterations in bone mass and in cortical and trabecular bone microarchitecture of long bones. Single-cell RNA sequencing (scRNA-Seq) of sensory neurons and bone tissue was next utilized to reconstruct potential nerve-to-bone signaling interactions, including implication of sensory nerve-derived neurotrophins (*Bdnf)*, neuropeptides (*Gal, Calca* and *Calcb*), and other morphogens (*Vegfa, Pdgfa*, and *Angpt2)*. Moreover, scRNA-Seq identified marked shifts in periosteal cell transcriptional changes within HFD-fed conditions, including a reduction in cell proliferation, an increase in adipogenic differentiation markers, and reductions in WNT, TGFβ, and MAPK signaling activity. When isolated, periosteal cells from HFD-fed mice showed deficits in proliferative and osteogenic differentiation potential. Moreover, these cellular changes in proliferation and differentiation capacity were restored by treatment of HFD-exposed periosteal cells to sensory neuron-conditioned medium. In summary, HFD modeling of type 2 diabetes results in skeletal polyneuropathy. Moreover, the combination of multi-tissue scRNA-Seq and isolated in vitro studies strengthen the case for altered nerve-to-bone signaling in diabetic bone disease.

## Intrduction

Diabetes is well established to cause end organ damage to both skeletal and neuronal systems.^1–4^ Up to 50% of patients with diabetes develop polyneuropathy, characterized by a length-dependent neuropathy with axonal degeneration, loss of distal fibers, and segmental demyelination.^5^ Symptoms typically include pain, paresthesias, and loss of sensation.^6,7^ Diabetes also causes prominent changes in the bone microenvironment, affecting osteoclast activity, bone resorption, and the function of skeletal progenitor cells.^8,9^ Research indicates that patients with diabetes have an elevated risk of hip, humerus, and foot fractures, particularly when accompanied by peripheral neuropathy.^10–12^ For example, in multivariate models, fracture risk in patients with diabetes was strongly associated with the presence of peripheral neuropathy.^11,12^ Likewise, an analysis of 2.8 million Veterans showed that older male Veterans with diabetes have a significantly increased risk of fracture, which was highly correlated to the presence of peripheral neuropathy.^13^ On one hand, co-occurrence of end organ damage in neural and skeletal systems may reflect the severity of diabetes. On the other hand, these aggregate clinical data suggest the possibility that diabetic neuropathy may represent a direct contributing factor in diabetic bone disease.

The crosstalk between peripheral afferent nerve fibers and bone has become a focus of recent research efforts. Studies have revealed that bone is richly innervated, particularly in the periosteum, which harbors a complex neural network with a high density of nerve fibers.^14–19^ Recent studies aimed at understanding the interaction between peripheral nerves and bone revealed a sophisticated interplay where peripheral nerves release various signaling molecules such as neurotransmitters, neuropeptides, and neurotrophins within the bone microenvironment.^18^ Moreover, sensory nerves that innervate the skeleton were shown to have a crucial role in the healing process of bone injuries in adult mice.^20^ However, there remains limited understanding regarding diabetic peripheral neuropathy associated with bone health and its connection to the cellular and signaling mechanisms involved in diabetic bone disease.

This study aimed to explore the relationship between diabetic neuropathy and diabetic bone disease using a mouse model fed a high-fat diet (HFD). Following the confirmation of fasting hyperglycemia and insulin resistance, further analysis evaluated skeletal innervation, bone microarchitecture and transcriptional changes by single-cell RNA sequencing (scRNA-Seq). These findings lend further credence to the notion that impaired nerve-to-skeletal cell paracrine signaling contributes to the pathophysiology of diabetic bone disease.

## Results

### HFD-feeding results in diabetic peripheral neuropathy

**Fig. 1:**
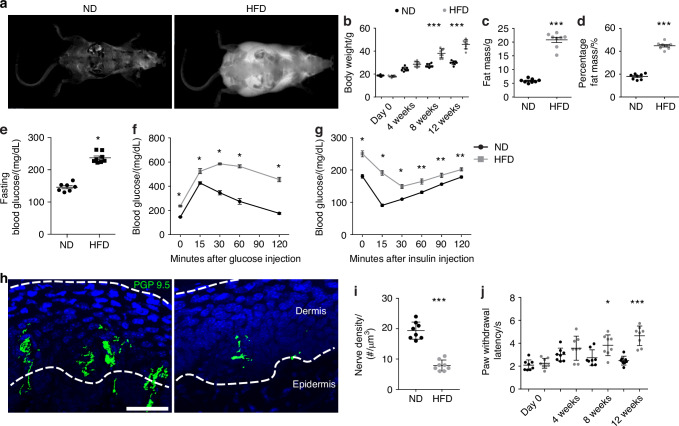
High-fat diet (HFD) feeding induces obesity, glucose intolerance, insulin resistance, and peripheral neuropathy. Normal diet (ND) or HFD feeding in C57BL/6J mice was instituted on week 4 of life, with analysis up to week 16 of life. **a** Representative Dual-energy X-ray absorptiometry (DXA) images of body fat. **b** Body weight after 0, 4, 8 and 12 weeks dietary treatment. *n* = 8 mice per group. **c** Fat mass and **d** Percentage of fat mass after 12 weeks dietary treatment. *n* = 8. **e** Fasting blood glucose after 12 weeks feeding. *n* = 8. **f** Blood glucose concentrations 0–120 min after glucose injection (1 g/kg body weight ip) in fasted mice following 12 weeks dietary treatment. *n* = 8. **g** Blood glucose concentrations 0–120 min after insulin administration (0.5 unit/kg ip) in fasted mice following 12 weeks dietary treatment. *n* = 8. **h**, **i** Images and quantification of Protein Gene Product 9.5 (PGP 9.5) immunofluorescent staining (green) in sagittal sections of mice hind paw skin after 12 weeks dietary treatment. *n* = 5. Scale bar: 100 µm. **j** Paw thermal withdrawal latency measurements at 0, 4, 8 and 12 weeks of dietary treatment. *n* = 8. Graphs represent average values ± 1 SD, *P < 0.05, **P < 0.01, and ***P < 0.001. Comparisons between groups were analyzed by unpaired Student’s *t* test. In subfigures (**b** and **j**), comparisons between groups were analyzed versus D7

C57BL/6J mice were fed a high-fat diet (HFD) over a 12 weeks period (from 4 to 16 weeks old). DXA analysis of body composition demonstrated increased body weight, fat body mass and percentage of fat mass over animals fed a normal diet (ND (Fig. 1a–d, 1.5-, 4- and 2.2-fold increase, respectively).^21,22^ Next, serologic studies were performed to confirm the induction of glucose metabolic dysfunction (Fig. 1e–g).^22^ Fasting blood glucose was significantly elevated in HFD-fed animals (Fig. 1e), and the HFD-fed cohort demonstrated glucose intolerance (Fig. 1f) and insulin resistance (Fig. 1g). The presence of a small peripheral neuropathy was first assessed by quantitative analysis of intraepidermal nerve fiber (IENF) density and behavioral paw withdrawal testing^23^ (Fig. 1h–j). Quantification of the immunostaining for the pan-neural marker protein gene product 9.5 (PGP 9.5) revealed a 32% reduction in IENF density in the HFD-fed cohort (Fig. 1i), concomitant with a significantly delayed paw withdrawal time during a hotplate test^24^ (Fig. 1j). These results confirmed the development of a type 2 diabetic polyneuropathy in HFD-fed mice.^25^

### Diabetic peripheral neuropathy affects long bone periosteum

**Fig. 2:**
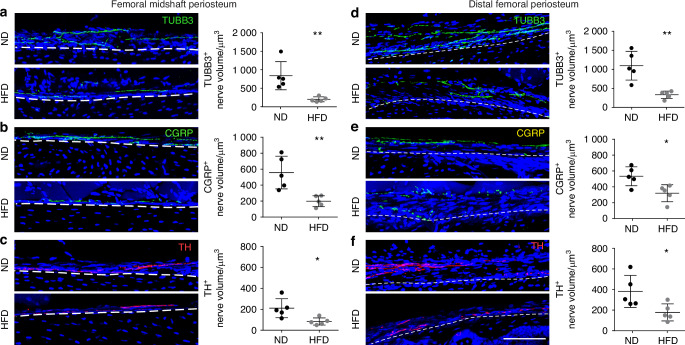
High-fat diet (HFD) feeding induces long bone neuropathy of the periosteum. Normal diet (ND) or HFD feeding in C57BL/6J mice was instituted on week 4 of life, with analysis at week 16 of life. **a** The innervation of the femoral midshaft periosteum visualized through the pan-neural marker β III-tubulin (TUBB3) immunostaining, represented in green. **b** Sensory innervation in the same region highlighted using Calcitonin gene-related peptide (CGRP) immunostaining, shown in yellow. **c** Sympathetic innervation observed through Tyrosine hydroxylase (TH) immunostaining, shown in red. *n* = 5. (Scale bar: 100 µm). **d** The distal femoral periosteum exhibits similar patterns for TUBB3^+^ total innervation, **e** CGRP^+^ sensory innervation and **f** TH^+^ sympathetic innervation. *n* = 5. Scale bar: 100 µm. Graphs represent average values ± 1 SD, *P < 0.05 and **P < 0.01. Comparisons between groups were analyzed by unpaired Student’s *t* test

Diabetic polyneuropathy is well-known to affect cutaneous innervation,^26^ but to our knowledge there are no reports on skeletal innervation in the context of type 2 diabetes. As the most innervated skeletal location,^27^ the long bone periosteum was evaluated. Nerve density within the periosteum was quantified within three representative bones, including the femur, tibia and the 1^st^ metatarsus. Immunostaining for the pan-neuronal marker β III-tubulin (TUBB3) was used to quantify total innervation. Calcitonin gene-related peptide (CGRP) and tyrosine hydroxylase (TH) were used to quantify sensory and sympathetic nerve fibers, respectively. Quantification of TUBB3^+^ nerve fibers showed a 76% decrease at the femoral midshaft under HFD-fed conditions (Fig. 2a). CGRP^+^ (Fig. 2b) and TH^+^ (Fig. 2c) nerve fibers also decreased by 62% and 55%, respectively. The distal femur periosteum was also assessed for total innervation, as well as sensory and sympathetic innervation. The results revealed similar significant decreases in TUBB3^+^ (Fig. 2d), CGRP^+^ (Fig. 2e) and TH^+^ (Fig. 2f) nerve fibers by 69%, 38% and 51%, respectively. The innervation at the tibial bone was similarly affected by HFD-feeding. Quantitative evaluation of periosteal innervation showed a 67% reduction of TUBB3^+^ nerve fibers at the midshaft and a 58% reduction at the distal tibia (Fig. S1). The innervation at the 1^st^ metatarsus was also affected in HFD-fed mice. Quantitative evaluation of periosteal innervation showed 43% and 41% at the midshaft and distal metatarsal periosteum, respectively (Fig. S2). These findings indicated that HFD-induced diabetic polyneuropathy extended to skeletal innervation affecting both more proximal long bones and distal small tubular bones of the feet.

### Bone alterations are associated with diabetic peripheral neuropathy

**Fig. 3:**
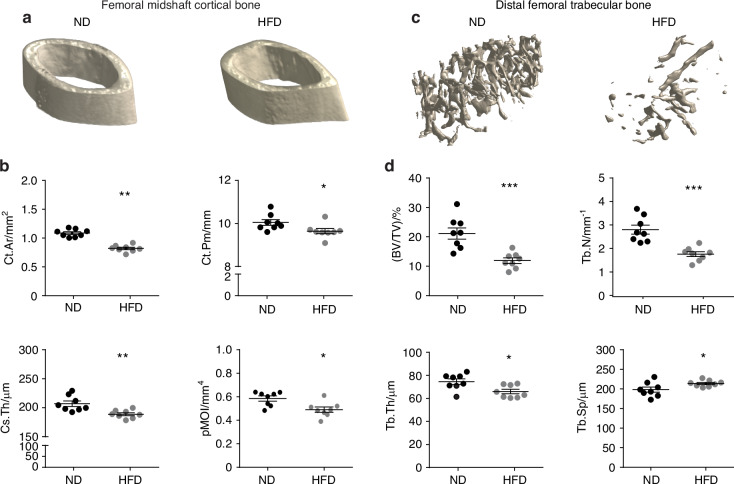
High-fat diet (HFD) feeding results in cortical and trabecular bone alterations. Normal diet (ND) or HFD feeding in C57BL/6J mice was instituted in week 4 of life, with analysis at week 16 of life. **a** µCT images of femoral midshaft cortical bone. **b** µCT quantifications of cortical area (Ct.Ar), cortical perimeter (Ct.Pm), cross-sectional thickness (Cs.Th) and polar moment of inertia (pMOI). *n* = 8 mice per group. **c** µCT images of distal femoral trabecular bone. **d** µCT quantifications of Bone volume per total volume (BV/TV), Trabecular thickness (Tb.Th), Trabecular number (Tb.N) and trabecular separation (Tb. Sp). *n* = 8 mice per group. Graphs represent average values ± 1 SD, *P < 0.05, **P < 0.01 and ***P < 0.001. Comparisons between groups were analyzed by unpaired Student’s *t* test

Next, quantitative analysis of cortical and trabecular bone in the femur, tibia and 1^st^ metatarsus were analyzed with microCT (Fig. 3 and Figs. S3–S5). Quantitative analysis of the microstructural parameters of the mid-shaft cortical bone including cortical bone area (Ct.Ar), cortical bone perimeter (Ct.Pm), cross-sectional thickness (Cs.Th) and polar moment of inertia (pMOI) were significantly decreased among HFD-fed mice showing a 11.9%, 4%, 8.3%, and 16.3% reduction in femur, respectively (Fig. 3a, b). Similarly, significant reductions in these parameters were observed in both the tibia and the 1^st^ metatarsus (Figs. S4a, b, S5a, b). For trabecular bone in the femur, fractional bone volume (BV/TV), trabecular thickness (Tb.Th), and trabecular number (Tb.N) were reduced by 42.6%, 11.4%, and 35.1%, respectively (Fig. 3c, d). These reductions were also evident in the tibia and the 1st metatarsus (Figs. S4c, d and S5c, d). Additionally, trabecular spacing (Tb.Sp) increased by 7.7% in the femur (Fig. 3d), with similar increases observed in the tibia and the 1^st^ metatarsus (Figs. S4d and S5d) within the HFD-fed cohort.

These quantitative analyses were further utilized to compare changes in cortical and trabecular bone parameters among the femur, tibia, and 1^st^ metatarsal bone. The comparative analysis revealed that reductions in cortical bone parameters were more pronounced in the more distally located small tubular bones of the feet (Table S3). In contrast, changes in trabecular bone parameters did not exhibit substantial differences between bones (Table S4).

### Multi-tissue single cell RNA-Sequencing infers somatosensory neuron-to-periosteal signaling

**Fig. 4:**
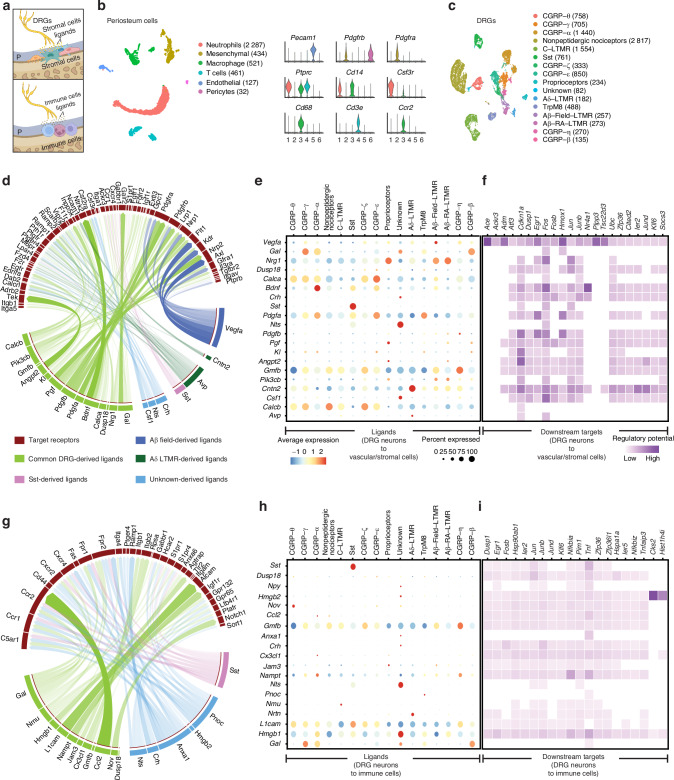
Cellular communication by interactome analysis between DRG neurons and long bone periosteum. **a** Schematic illustrating the interaction between dorsal root ganglia (DRG) sensory neurons and periosteal cells within their microenvironment. **b** UMAP projection of periosteum cell clusters via single-cell RNA sequencing (scRNA-Seq), accompanied by violin plots of known gene markers for each cluster. *n* = 4 042 total cells. Cell number per cluster shown in parentheses. **c** UMAP visualization of mouse lumbar dorsal root ganglia (DRG) neurons by scRNA-Seq, re-analyzed from ref. ^102^ *n* = 1 757 total DRG neurons. Cell number per cluster shown in parentheses. **d** Interaction plot depicting the cell-cell communication between DRG neurons and periosteal stromal/vascular cells (including mesenchymal cells, endothelial cells, and pericytes). DRG neuron-derived ligands and receptors expressed on stromal/vascular cells are shown under normal diet conditions. *n* = 593 stromal/vascular cells. **e** Expression profile of ligands in each DRG neuron cell cluster, highlighting specificity to stromal/vascular cells. **f** Analysis of the regulatory potential of DRG neuron-derived ligands and their downstream target genes in stromal/vascular cells. **g** Expression profile of ligands in each DRG neuron cell cluster and corresponding receptors within immune cells of the periosteum (including macrophage, neutrophils and T cells). *n* = 3 269 immune cells. **h**, **i** Analysis of the regulatory potential of DRG neuron-derived ligands and their downstream target genes in immune cells

Communication between sensory nerve and bone cells has been demonstrated at a cellular level, forming a neuro-osteogenic network within the periosteum.^28,29^ We next set out to decipher potential intercellular communication between DRG sensory neurons and skeletal resident cells within the periosteum (Fig. 4a). Long bone periosteum (combined femoral and tibial periosteum) was isolated and examined by scRNA-Seq analysis in ND conditions. Six cellular clusters were identified, including mesenchymal cells (*n* = 434), endothelial cells (*n* = 127), pericytes (*n* = 32), neutrophils (*n* = 2 287), macrophages (*n* = 521), and T cells (*n* = 461) (Fig. 4b). A previously established scRNA-Seq dataset from mouse lumbar DRG neurons was reanalyzed, with 16 identified neuronal subclusters^30^ (Fig. 4c). These included 7 subclusters of CGRP neurons, low threshold mechanosensory neurons (LTMRs), nonpeptidergic nociceptors, proprioceptors, somatostatin^+^ (SST) neurons, and transient receptor potential M8 (TRPM8) cold sensitive neurons. Next, interaction analyses between DRG neurons and periosteal cells was performed using NicheNet (Fig. 4d–f),^31^ for which, cells from periosteum were defined as receiver cells and categorized into two distinct niches: the periosteal stromal/vascular niche (mesenchymal cells, endothelial cells, and pericytes) and the periosteal immune cell niche (neutrophils, macrophages, and T cells). With the neuronal derived ligands as senders and receiver cells defined, a prediction for ligand-receptor interactions was performed by combining gene expression with existing knowledge of signaling pathways and gene regulatory networks. The goal was to identify predicted neuronal ligands secreted from DRG neurons and their downstream signaling genes involved specifically in periosteum cellular niche regulation.^31^

The ligand-receptor interaction analysis between DRG neurons and the periosteum stromal/vascular niche revealed enriched expression of numerous neural ligands, including growth factors (*Vegfa*, *Nrg1*, *Gmfb, Pdgfa* and *Pdgfb*) and neuropeptides (*Gal*, *Calca*, *Bdnf*, and *Calcb*), predicted to pair with stromal/vascular cell receptors^32,33^ (Fig. 4d). Among the growth factors, Vascular Endothelial Growth Factor A (VEGFA) was highly expressed by Aβ−Field−LTMR neurons, a critical regulator of osteoblast function, bone homeostasis and skeletal repair.^15,34–38^ The membrane glycoprotein Neuregulin 1 (NRG1), was highly expressed across multiple DRG neuron types, including proprioceptors, Aβ−Field−LTMR and Aβ–RA−LTMR neurons. NRG1 has been implicated during osteoblastogenesis^39^ and in cartilage and bone regeneration in zebrafish.^40^ Another growth and differentiation factor enriched in the interaction analysis was Glia Maturation Factor Beta (GMFB). *Gmfb* was highly expressed in nonpeptidergic nociceptors, CGRP-ζ and CGRP-η neurons. A recent study showed that *Gmfb* knockout mice are protected against osteoporosis by suppressing osteoclast hyperactivity in diabetic conditions.^34^ Platelet-derived growth factors A and B (PDGFA and PDGFB) were also identified as key soluble growth factors in the interaction analysis. *Pdgfa* exhibited high expression in CGRP α, ε, and Trpm8 neurons, while *Pdgfb* was most highly expressed in CGRP-η neurons. PDGFA has been implicated in nerve-regulated digit tip regeneration,^41^ and our group implicated its potential role as a nerve-derived factor in regulation of heterotopic ossification (HO).^42^ Galanin (GAL) was highly expressed by CGRP-γ, α and β neurons. Several studies showed that Galanin (GAL) was present in bone marrow mesenchymal cells, endothelial cells and nerve fibers within the periosteum suggesting a role in bone development and repair. *Calca* and *Calcb* genes encoding Calcitonin gene-related peptide (CGRP), a crucial neuropeptide for bone metabolism regulation, were also enriched in the interaction analysis, primarily in CGRP clusters. CGRP was shown to play a pleiotropic effect on bone cells, promoting osteoblast differentiation, inhibiting osteoclast activity,^43^ and enhancing reparative bone formation in response to mechanical loading and fracture.^44^ Brain-Derived Neurotrophic Factor (BDNF) was also enriched in the interaction analysis. *Bdnf* was highly expressed by CGRP-α, Sst and CGRP-ε neurons. Several studies have shown the positive effects of BDNF on bone formation by enhancing osteoblast differentiation, new bone formation and maturation.^45,46^ Moreover, BDNF and its receptor TrkB are present at various stages of the bone formation process and are upregulated in human osteoblasts, suggesting a role in bone development^47,48^ (Fig. 4e). Although the interaction analysis did not predict previously described bone-related neural ligands such as Fibroblast growth factor 1 and 9 (FGF1, FGF9),^49–51^ Follistatin Like 1 (FSTL1)^17^ and Sonic hedgehog signaling molecule (SHH),^52^ their expression was present within the present dataset of DRG neurons.

Following up the ligand-receptor interaction, various downstream signaling events were predicted to regulate target genes involved in cell cycle regulation (*Cdkn1a* and *Dusp1*), cell growth and proliferation (*Ackr3*, *Ergr1*, *Fosb* and *Socs3*), cell differentiation (*Fos*, *Fosb* and *Ier2*) and transcriptional regulation (*Jun*, *Junb*, *Jund*, *Nr4a1* and *Cited2*). Interestingly, several downstream target genes were described to play an important role in regulating osteoblast differentiation and bone formation among them, Fos and Jun family members,^53^ the zinc finger protein *Zfp36*,^54^ the heme oxygenase *Hmox1*,^55^ the cyclin-dependent kinase inhibitor *Cdkn1a*,^53^ the suppressor of cytokine signaling *Socs3*,^56^ and the nuclear receptor *Nr4a1*^53^ (Fig. 4f).

Next, a similar interaction analysis was performed between DRG neurons and immune periosteal niche (including neutrophils, macrophages, and T cells). The ligand-receptor interaction analysis revealed enriched expression of numerous neural ligands including, neuropeptides (*Nrtn*) and neurotransmitters (*Sst*, *Pnoc*, *Nmu*, and *Gal*), cell adhesion molecules (*l1cam*), cytokines and chemokines (*Ccl2* and *Cx3cl1*) predicted to pair with immune cells receptors (Fig. 4g). Neurturin (*Nrtn*) gene was highly expressed by Aδ-LTMR neurons. It has been suggested that NRTN may play several context-dependent roles in modulating immune cell function and inflammation.^57^ Somatostatin (SST), a neurotransmitter known for the neuroendocrine inhibitory effects across multiple systems^58^ was mainly expressed by the Sst neurons. Sst is known to exert inhibitory effects on immune cell functions, cytokine production, proliferation, and inflammatory responses.^59,60^ Another neurotransmitter, Prepronociceptin (*Pnoc*) gene was specifically expressed by TrpM8 neurons. PNOC is the precursor protein for nociceptin, a neuropeptide involved in regulating inflammation, pain, and arousal.^61^ Neuromedin U gene (*Nmu*) was specifically expressed by C-LTMR neurons. Nmu is an immunoregulator described to be a potent activator of immune cells, particularly in the context of type 2 inflammation, allergic responses, and autoimmune conditions.^62,63^ Galanin gene (*Gal*) was highly expressed by CGRP-γ, CGRP-α, and CGRP-β neurons. *Gal* was described to modulate the expression of cytokines suggesting a role in regulating inflammatory responses.^64,65^ The L1 Cell Adhesion Molecule gene (*l1cam*) was highly expressed in CGRP-θ and Sst neurons. L1CAM was shown to contribute to the migration and extravasation of immune cells.^66^ Chemokines and cytokines genes such as C-C Motif Chemokine Ligand 2 (*Ccl2*) and C-X3-C Motif Chemokine Ligand 1 (*Cx3cl1*) were highly expressed in CGRP-θ and CGRP-α, respectively. CCL2 was shown to be a key mediator of the crosstalk between immune and bone cells, influencing bone homeostasis^67,68^ (Fig. 4h). Interestingly, some ligands, such as SST, DUSP18, CRH, NTS, GAL and GMFB, were found to activate both mesenchymal and immune cell populations, suggesting shared signaling pathways in the periosteal niche. The ligand-receptor analysis was followed by a downstream signaling inquiry highlighting the activated genes within the periosteal immune niche involved in cell cycle regulation (*Cks2* and *Dusp1*), cell growth and proliferation (*Erg1*, *Fosb*, *Jun* family and *Pim1*), cell differentiation (*Klf6* and *Zfp36*), and immune response (*Nfkbi* family and *Tnf*) (Fig. 4i).

The analysis model presented here describes secreted neural ligands regulating downstream genes under homeostatic conditions. Interestingly, distinct sets of ligands were predicted to activate downstream genes in periosteal mesenchymal cells versus periosteal immune cells, predicted to regulate cell cycle and proliferation, as well as cell function.

### HFD-induced bone neuropathy disturbs long bone periosteal cellular signalization

**Fig. 5:**
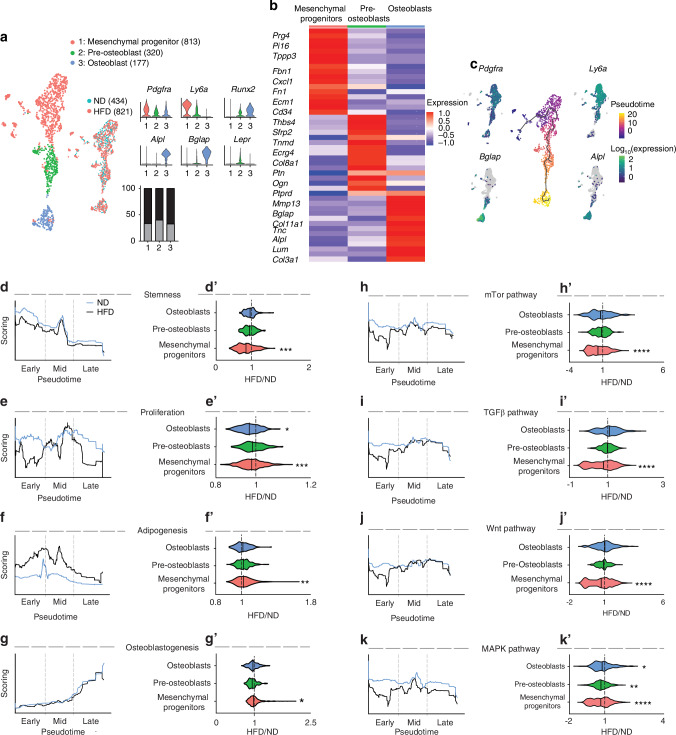
High-fat diet (HFD) feeding disturbs the skeletal cell phenotype and neural-skeletal cell communication within the periosteal microenvironment. 12 weeks after the initiation of the dietary treatment, four left femurs and tibias from ND and HFD mice were dissected. Periosteal cells were isolated, and scRNA-sequencing and analysis were performed. **a** UMAP of mesenchymal cell subclusters including mesenchymal progenitors, pre-osteoblasts, and osteoblasts, along with known gene markers for each by violin plot, and the cell number ratio among ND and HFD treated groups. Black indicates ND cells. Gray indicates HFD cells. **b** Heatmap for differentially expressed genes (DRGs) in each mesenchymal cell subcluster. **c** UMAP showing the pseudotime trajectory of the mesenchymal cell subclusters along with progenitor gene markers (*Pdgfrα* and *Ly6a*) as well as osteoblast markers (*Bglap* and *Alpl*). Black line represents the trajectory graph. Plots were generated using Monocle 3.0.1.2. **d**–**g’** Linear graph analysis of phenotype changes across pseudotime including gene modules for stemness, proliferation, adipogenesis and osteoblastogenesis among ND (blue line) and HFD (black line) fed mesenchymal cells, and module index scoring of mesenchymal cell subclusters (mesenchymal progenitors, pre-osteoblasts and osteoblasts). Dashed lines indicate delineates early, mid and late pseudotime. **h**–**k’** Linear graph analysis of dysregulated signaling pathways, including MAPK, TGFβ, Wnt and mTor signaling, across pseudotime among ND (blue line) and HFD (black line) fed groups and module index scoring of mesenchymal cell subclusters. Dashed gray lines in module score graphs represent early, mid, and late pseudotime. Graphs represent average values ± 1 SD. Module scoring data was analyzed using the Kolmogorov-Smirnov test. *P < 0.05, **P < 0.01, ***P < 0.001 and ****P < 0.00 01 in comparison to ND control. 1 255 total mesenchymal cells analyzed

Next, scRNA-Seq analysis focused on signaling changes in the transcriptome among periosteal cells exposed to either ND or HFD-feeding conditions (Fig. 5 and Fig. S6). First, changes in the cellular composition of the six periosteal cell clusters (Fig. S6a), were analyzed, revealing subtle differences in the distribution of clusters between ND and HFD conditions (Fig. S6b, c). Next, overall changes in cellular proliferation were assessed using a proliferation module score ratio (HFD/ND) calculated across periosteum cell clusters (Fig. S6d). A significant reduction in proliferative score was seen under HFD conditions among several cell types including pericytes, T cells and mesenchymal cells (Fig. S6d). Kyoto Encyclopedia of Genes and Genomes (KEGG) enrichment analysis using all periosteal cells highlighted increased terms expression under ND conditions including ossification and angiogenesis, as well as signaling pathways such as mitogen-activated protein kinase (MAPK), Vascular endothelial growth factor (VEGF), and Transforming growth factor beta (TGFβ). Conversely, representative GO terms enriched within the HFD periosteal cells included bone resorption, hypoxia, negative regulation of angiogenesis, and cellular immune response (Fig. S6e). Further transcriptional analysis focused on the mesenchymal cell cluster, dissecting their cell subcluster frequency, phenotypes, and molecular signaling pathway modifications associated to HFD feeding conditions (Fig. 5). Mesenchymal cells were analyzed and distributed across three subclusters defined by characteristic gene markers, subcluster 1: mesenchymal progenitor cells (*Pdgfrα*^*+*^*Ly6a*^*+*^, *n* = 813), subcluster 2: Pre-osteoblasts (*Ly6a*^*+*^*Runx2*^*+*^*Lepr*^*+*^, *n* = 320), and subcluster 3: Osteoblasts (*Runx2*^*+*^*Alpl*^*+*^*Bglap*^*+*^, *n* = 177) (Fig. 5a). Next, characteristic gene markers were examined across the three subclusters as shown by heatmap of differentially expressed gene (DEG) profiles (Fig. 5b). Complete DEGs are provided in (Fig. S7) along with characterize periosteal cell markers such as *Gli1*,^69^ *Postn*,^70^ *Ctsk*,^71^ *Acta2*^72^ and *Nes*.^73^

Next, pseudotime trajectory analysis revealed a continuous cell differentiation path,^74^ starting from a mesenchymal progenitor cell expressing *Pdgfrα*^*+*^ and *Ly6a*^*+*^ genes. As cells progressed along the pseudotime axis, they transitioned through an intermediate pre-osteoblast state, characterized by the expression of *Ly6a*, *Runx2*, and *Lepr* genes. The terminal branch represented fully differentiated osteoblast cells, expressing *Runx2*, *Alpl*, and *Bglap* (Fig. 5c).^75^ Here, the trajectory analysis laid the foundation for further investigation of the phenotypical changes and regulatory signaling under ND and HFD-fed conditions. Using the R package Escape,^76^ we evaluated the gene expression changes involved in phenotypical programs and signaling pathways across pseudotime, dividing them into early, mid, and late pseudotime stages. To further quantify changes in gene module scores, we employed violin plots, which allowed us to assess these changes among mesenchymal progenitor cells, pre-osteoblasts, and osteoblast cell clusters (Fig. 5d–k’). Phenotypical changes across pseudotime analysis in gene module scores related to stemness, proliferation, adipogenesis and osteoblastogenesis were first assessed (Fig. 5d–g’). Periosteal cells from ND exposed animals exhibited higher gene modules scores related to both stemness and proliferation in comparison to cells from HFD exposed animals, particularly in early pseudotime and among mesenchymal progenitor cells (Fig. 5d, e’).

Gene module scores related to adipogenesis were increased across pseudotime in HFD derived cells, which although elevated across pseudotime achieved statistical significance in mesenchymal progenitor cells (Fig. 5f, f’). In contrast, gene module scores related to osteoblastogenesis were similar between experimental conditions until late pseudotime, potentially indicating impaired terminal osteoblast maturation under HFD conditions (Fig. 5g, g’).

Signaling pathway changes across pseudotime and by mesenchymal cell subcluster was next assessed (Fig. 5h–k’, Fig. S8). Interestingly, numerous signaling pathways showed alteration in mesenchymal cells derived from HFD conditions, and much of these changes were most evident in the more stem-like mesenchymal cell subcluster. Those with highest predicted derangement are shown in Fig. 5h–k’, and include mammalian target of rapamycin (mTOR), TGFβ, and Wnt pathways which were decreased in early pseudotime among HFD exposed mesenchymal progenitor cells (Fig. 5h–j’). The MAPK signaling pathway was the most dysregulated pathway under HFD conditions, showing significant reduction across pseudotime and within each mesenchymal cell subcluster (Fig. 5k, k’). Other signaling pathways likewise showed moderate reductions in morphogenic signaling under HFD conditions (Fig. S8), including VEGF and Notch pathways, which exhibited a decrease in early pseudotime among HFD-exposed mesenchymal progenitor cells. Additionally, Hedgehog and Jak-Stat pathways demonstrated a decrease in both early and late pseudotime within the mesenchymal progenitor subcluster. As for FGF signaling, an initial decrease in early pseudotime was followed by an upward trend to finally decrease significantly at the latest pseudotime. BMP signaling conspicuously decreased in mid pseudotime among HFD exposed mesenchymal progenitor cells.

Thus, HFD- feeding and consequent neuropathic and osteopathic changes are prominently observable at the single-cell transcriptional level within long bone periosteum, and include a transcriptional signature suggesting impaired stemness, proliferation, and differentiation potential associated with myriad signaling pathway changes.

### DRG neuron conditioned media restores the proliferation and differentiation capabilities of HFD-exposed periosteal cells

**Fig. 6:**
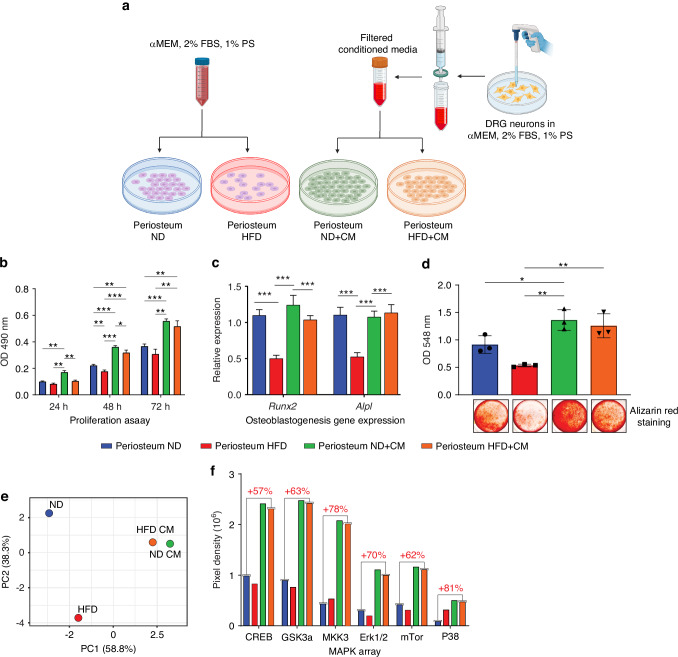
DRG neural conditioned media effects on periosteal cells after dietary intervention. **a** Schematic representation of mouse DRG neuron-derived conditioned medium (CM) and treatment of mouse periosteal cells derived from ND or HFD dietary treatment for 12 weeks. **b** Cell proliferation assay of periosteum mesenchymal cells isolated from ND or HFD-fed animals after 24, 48 and 72 h treated with DRG neuron-derived CM or control media. **c** Gene expression for osteogenic markers Runt-related transcription factor 2 (*Runx2*) and Alkaline Phosphatase (*Alpl*) after 72 h treatment with or without CM. **d** Quantification and staining for alizarin red 2 weeks after osteogenic differentiation with or without CM treatment. **e** PCA analysis of total protein extracted 2 wks after osteogenic differentiation with or without CM treatment. **f** Quantification of individual proteins from MAPK protein array. *n* = 3 per each group in the in vitro experiment. Graphs represent average values ± 1 SD. Two-tailed ANOVA with post-hoc analysis was performed between the four experimental groups. *P < 0.05, **P < 0.01 and ***P < 0.001

Having identified a skeletal neuropathy in diabetic conditions and implicated signaling pathways alterations in nerve-to-bone interactions, we next sought to validate these findings using isolated in vitro experimentation (Fig. 6). Freshly harvested periosteal mesenchymal cells were isolated from C57BL/6J mice exposed to either ND or HFD-feeding conditions for 12 weeks. These cells were either exposed to control medium or to conditioned media (CM) obtained from healthy mouse DRG neurons (Fig. 6a). Cell proliferation was first assessed (Fig. 6b). After 24 h of culture, the proliferation assay revealed an expected decrease in proliferation for periosteal cells^77^ from animals exposed to HFD compared to ND conditions. Supplementation of neuronal conditioned media (CM) led to enhanced proliferation for ND periosteal cells as well as restoration of proliferation for HFD periosteal cells, compared to respective controls. These outcomes were consistently replicated after 48 h and 72 h (Fig. 6b). Next, osteogenic differentiation was induced in ND and HFD periosteal cells with or without neural CM treatment. RT-qPCR analysis on the periosteal cells from animals exposed to HFD compared to ND conditions showed a decrease in differentiation marker *Runx2* and *Alpl* (Fig. 6c, day 21 of differentiation). Neural CM did not significantly increase expression levels in ND exposed periosteal cells but restored the expression of these markers in HFD periosteal cells. Consistent with gene expression findings, Alizarin red staining revealed a decrease in bone nodules in periosteal cells from animals exposed to HFD as compared to ND conditions.^78^ Neural CM treatment led to an increase in bone nodule deposition among ND exposed cells, and restored bone nodule deposition within HFD periosteal cells (Fig. 6d). Among all signaling pathways examined transcriptomically, MAPK signaling module scores showed the broadest derangement across pseudotime and among each mesenchymal subcluster in response to HFD. This insight prompted an examination of the effects of neural CM on MAPK signaling phosphorylation and activation among ND and HFD derived periosteal cell cultures (Fig. 6e, f). A MAPK phospho-protein array was performed after 72 h of culture with or without neural CM treatment among ND and HFD derived periosteal cells.^79^ Principal Component Analysis (PCA) of total protein extracted showed a distinct grouping of samples based on dietary treatment (Fig. 6e). Quantification of MAPK phosphorylated proteins revealed a significant decrease in MAPK signaling proteins including CAMP Responsive Element Binding Protein 1 (CREB), Glycogen Synthase Kinase 3 Alpha (GSK3a), extracellular signal-regulated kinases 1/2 (Erk1/2) and mTor under HFD conditions. Neural CM treatment led to robust increases in phospho-MAPK signaling, with the highest fold increases observed in CREB, GSK3a, MKK3, Erk1/2 and mTor expression in both ND and HFD periosteal cells when exposed to neural CM (Fig. 6f and Fig. S9). Thus, periosteal cells from HFD-fed mice demonstrate deficits in proliferative and osteogenic differentiation potential. Importantly, these cellular changes in proliferation, differentiation and signaling activity were restored by treatment of HFD-exposed periosteal cells to sensory neuron conditioned medium.

## Discussion

This study demonstrated that high-fat diet (HFD)-induced type 2 diabetes leads to peripheral neuropathy affecting the innervation of long bones, particularly the periosteum. The analysis of long bone microarchitecture revealed a reduction in both trabecular and cortical bone mass under HFD conditions. The computational model employed in this study deciphered the intricate communication between somatosensory neurons and the periosteal cellular niche, highlighting the crucial role of this neuro-osteogenic circuitry in regulating bone homeostasis.

Peripheral neuropathy has many potential causes, with diabetic peripheral neuropathy (DPN) being the most common subtype. DPN can result in severe complications, from paresthesia to limb loss. While the precise cause of DPN remains unknown, theories involving metabolic, neurovascular, and autoimmune pathways have been suggested. In this study, quantitative analyses revealed a significant reduction in intraepidermal nerve fiber density and delayed paw withdrawal time, confirming the development of diabetic polyneuropathy in HFD-fed mice. Furthermore, immunostaining for neuronal markers showed a substantial decrease in total, sensory, and sympathetic nerve fibers within the periosteum of the femur, tibia, and metatarsus, indicating that HFD-induced neuropathy extended to skeletal innervation. These findings are in line with other observations where, for example, bone marrow innervation was found to be reduced in a type 1 diabetes model.^80^

The intricate paracrine interactions between the somatosensory nervous system and bone is becoming increasingly evident.^33,81^ Here, we focused on potential neurotrophic/neurosecretory functions of the peripheral nervous system to long bone periosteum under homeostatic conditions. Several neural ligands were identified, including growth factors (VEGFA, NRG1, GMFB, PDGFA, and PDGFB) and neuropeptides (GAL, CALCA, BDNF, and CALCB), that are predicted to interact with receptors on periosteal stromal/vascular and periosteal immune cells. These ligands are known to play crucial roles in osteoblast and osteoclast function, bone homeostasis, and repair processes. Interestingly, some ligands, such as VEGFA and GMFB, were found to activate both mesenchymal and immune cell populations, suggesting shared signaling pathways across cell types within the periosteal niche. The identification of these neural ligands and their potential interactions with periosteal cells provides insights into the communication between the peripheral nervous system and bone, which may be disrupted in diabetic conditions.^37,48,82–86^ Of note, dysfunction in peripheral nerves have also been postulated to contribute to diabetic bone disease via abnormal bone loading (neurotraumatic hypothesis)^87^ and or abnormal nerve-to-vessel signaling (neurovascular hypothesis), and these concepts were not explicitly explored here.

The impact of high-fat diet (HFD) conditions on periosteal cell signaling pathways has significant implications for bone health.^88,89^ This study revealed dysregulation of diverse signaling pathways, including mTOR, TGFβ, Wnt, VEGF and MAPK, in periosteal cells under HFD conditions. Notably, the MAPK signaling pathway was prominently downregulated in response to HFD across mesenchymal cells and subclusters, and was seen by decreased phosphorylation of MAPK signaling molecules, such as CREB, GDK3a, mTor and ERK1/2 when isolated in culture. Recent research highlights the involvement of CREB, GSK3α, ERK1/2, and mTOR in the pathophysiology of diabetic bone disease. CREB, essential for glucose homeostasis and β-cell survival, is regulated by glucose-induced calcium entry and phosphorylation via ERK1/2, underscoring its role in diabetes management.^90^ While specific studies on GSK3α in diabetic bone disease are limited, GSK3 inhibition generally promotes osteogenic differentiation, potentially counteracting diabetes-induced bone loss.^91^ The ERK1/2 pathway exhibits a biphasic role in osteogenesis, promoting bone formation in precursor osteoblasts but inhibiting it in mature cells, and also modulates CREB phosphorylation, linking it to glucose-mediated β-cell survival. Additionally, the mTOR pathway, critical for cellular energy metabolism, interacts with ERK1/2 to regulate osteoblast function and energy metabolism, with its inhibition shown to reduce bone formation.^90,91^ The restoration of MAPK signaling and associated cellular processes upon exposure to neural conditioned media highlights the potential therapeutic implications of targeting this pathway in diabetic bone.

Several limitations to the above study deserve acknowledgement. First, the study utilized young animals (4–16 weeks old), and the effects observed may differ in older populations or with longer durations of diabetes. Second, the study focused solely on male mice, and potential sex-specific differences in the observed phenomena cannot be ruled out.^92–94^ This is specifically important in that men and women experience orthopedic pain differently, suggesting biological relevant sex differences in somatosensory pathways to bone.^95,96^ Finally, the neural conditioned media used in the in vitro experiments was derived from a mixed population of DRG neurons and non-neuronal support cells present within the ganglia, which may not with high fidelity represent the specific somatosensory neural signals present in the periosteal niche.

In summary, this study provides compelling evidence that HFD-induced type 2 diabetes leads to peripheral neuropathy affecting the innervation of long bones. The identification of neural ligands and their potential interactions with periosteal cells, as well as the dysregulation of signaling pathways, offer insights into the mechanisms underlying the observed bone alterations in diabetic conditions. While acknowledging the limitations of the study, these findings contribute to our understanding of the complex interplay between the nervous system, metabolism, and skeletal health, paving the way for potential therapeutic interventions targeting these pathways in diabetic bone disorders.

## Material and methods

### Animal use

All experiments were conducted under Johns Hopkins University ACUC approval. C57BL/6J male mice were purchased from Jackson Laboratories Bar Harbor, Maine. HFD feeding was instituted at 4 weeks of age and animals were maintained on normal diet (ND) chow or high-fat diet (HFD) chow containing 60% of calories from fat (Research Diets, New Brunswick, NJ; catalogue #D12492) for 12 weeks. Right femurs, tibias and 1^st^ metatarsal bones were harvested and fixed overnight in 4% paraformaldehyde at 4 °C and were used for micro-computed tomography (µCT) and histology. Muscles and connective tissues were removed from left femurs, tibias, and were digested to isolate periosteal cells. *n* = 5 male C57BL/6J mice were analyzed for periosteal nerve quantification, *n* = 8 for femoral and tibial microarchitecture analysis, and *n* = 6 for metatarsal microarchitecture analysis were used.

### Glucose tolerance test (GTT) and insulin tolerance test (ITT)

Tests were performed at 16 weeks of age, after 12 weeks of HFD or ND feeding. Mice were fasted for 6 h by removing food at 8 am, and tests were initiated at 2 pm. Fasting glucose was measured after the 6 h fast. Blood glucose levels were measured at 0, 15, 30, 60, 90 and 120 min after glucose (1 g/kg) i.p. injection and at 0, 15, 30, 60, 90 and 120 min after insulin (0.5 unit/kg) i.p. injection. Blood glucose levels were monitored from the tail-tip using a hand-held glucometer (Contour next EZ, Ascensia Diabetes Care US Inc, NJ, USA).

### Dual-energy X-ray absorptiometry (DXA) assessment of body composition

Measurement of body composition including body weight, fat body mass, and percentage of fat mass was assessed by DXA at baseline and every 4 weeks until the cessation of the feeding period using a UltraFocus Faxitron equipment (Faxitron Bioptics, Tucson, AZ).

### Micro-Computed Tomography (MicroCT) scans and analyses

MicroCT scans were performed using a SkyScan1172 high-resolution microCT imaging system (Bruker, Kontich, Belgium). Right femurs, tibias and the first metatarsus of mice were scanned at a resolution of 6 µm with the following settings: 1 mm of aluminum filter, X-ray voltage of 65 kVP, anode current of 153 uA, exposure time of 65 ms, frame averaging of 4, and rotation step of 0.3 degrees. Three-dimensional images were then reconstructed from the 2D X-ray projections by implementing the Feldkamp algorithm using a commercial software package NRecon software (2.0.4.0 SkyScan). For the 3D morphometric analyses of images, CTVox and CTAn software (1.13 SkyScan) were used. The following trabecular bone microarchitectural parameters were calculated: bone volume/total volume ratio (BV/TV, %), trabecular thickness (Tb.Th, µm), trabecular number (Tb.N, /mm^−1^), and trabecular separation (Tb.Sp, µm). Cortical bone parameters were determined from 50 slices at the femoral and tibial mid-shaft, and 20 slices at the metatarsal mid-shaft. The following parameters were calculated: cortical bone area (Ct.Ar, mm^2^), cortical perimeter (Ct.Pm, mm), cortical thickness (Ct.Th, µm), and polar moment of inertia (pMOI, mm^4^). The femur length was measured manually on microCT reconstructed images using CtAn software. Briefly, after realigning the femur perpendicularly, the distance between the distal and proximal epiphyses were measured by using the measurement tool available on CtAn.

### Histology and immunohistochemistry

Right femurs, tibias and the first metatarsal bones were harvested at the experiment endpoint and placed in 4% paraformaldehyde (PFA) at 4 °C for 24 h. After sequential washes in PBS x 3 for 20 min, samples were decalcified in 14% EDTA (Thermo Fisher Scientific, Waltham, MA) for 28 day at 4 °C. For cryosections, samples were cryoprotected in 30% sucrose overnight at 4 °C before embedding in OCT (Tissue-Tek 4583, Torrance, CA). Sagittal sections were obtained at 14 or 40 µm thickness. Sections were mounted on adhesive slides (Fisherbrand™ Superfrost™ Plus Microscope Slides, Fisher Scientific, Nazareth, PA). For immunohistochemistry, sections were washed in PBS x 3 for 10 min, and permeabilized with 0.5% Triton-X for 30 min. Next, 3% normal goat or donkey serum was applied for 60 min, then incubated in primary antibodies overnight at 4 °C in a humidified chamber (see Table S1 for antibodies used). The following day, slides were washed in PBS, incubated in the appropriate secondary antibody for 1 h at 25 °C, then mounted with DAPI mounting solution (Vectashield H-1500, Vector Laboratories, Burlingame, CA). Digital images were captured with 10–20× objectives using upright fluorescent microscopy (Leica DM6, Leica Microsystems Inc., Buffalo Grove, IL) or confocal microscopy (Zeiss LSM780 FCS, Carl Zeiss Microscopy GmbH, Jena, Germany). For immunostaining analysis, the surface plugin in Imaris software v9.3 (Oxford Instruments, Belfast, UK) was used, six random 20x three-dimensional volumetric regions of interest (200*1 000*40 pixels) were analyzed per sample which were centered around the periosteum. Data per sample is presented as a mean value of all six regions of interest. In all cases, the assessors were blinded to the different groups.

### DRG isolation, culture and conditioned media (CM) preparation

Lumbar (L1-L5) DRGs were harvested at the experimental endpoint and kept in cold αMEM with 10% FBS and 1x penicillin/streptomycin.^17^ DRGs were then digested with 1 mg/mL type I collagenase (Thermo Fisher Scientific) and 5 mg/mL dispase II (Thermo Fisher Scientific) in αMEM at 37 °C for 70 min, and dissociated into single cells by trituration with a 1-mL pipette tip. The dissociated cells were filtered with a 70-μm cell strainer and centrifuged at 500 rcf for 5 min. Cells were immediately mixed with pre-warmed αMEM with 5% FBS, 1x penicillin/streptomycin, 1x Glutamax (Thermo Fisher Scientific), and anti-mitotic reagents (20 μmol/L 5-fluoro-2-deoxyuridine and 20 μmol/L uridine, Sigma-Aldrich) and 10 000 cells were seeded into 12-well plates pre-coated with 100 μg/mL poly-D-lysine (Sigma-Aldrich) and 10 μg/mL laminin (Thermo Fisher Scientific). Next, to generate conditioned media (CM), DRG neurons were allowed to grow for 4–5 day to expand their axonal network. The media was then changed to αMEM with 1% FBS. Neural CM was harvested every day for 5 days, filtered (0.22 µm) and frozen at −80 °C until use. Unconditioned medium with the same base constituents was used as a control in all experiments.

### Periosteum progenitor cells isolation and culture

Three left femurs and tibias were harvested from ND and HFD groups at the experimental endpoint. The dissected bones were digested in 3 mg/mL collagenase I (Worthington), 4 mg/mL dispase II (Roche) in PBS for 6 × 15 min at 180 r/min at 37 °C.^97^ Samples were run through a 40 μm filter (VWR) and rinsed with 3 mL αMEM (Gibco) with 20% FBS. Cells were centrifuged at 300 × *g* for 30 min at 4 °C. The cell pellet was washed twice with 1 mL PBS and spun for 10 min at 300 × *g* at 4 °C. Cells were cultured in αMEM, 2 mmol/L GlutaMax, 10% FBS until they reached 75% confluence. An MTS assay was used to quantify cell proliferation at 24, 48 and 72 h of culture with or without neural CM treatment, based on the manufacturer’s protocol.^98,99^ A MAPK phospho-protein array was performed using C-Series Human/Mouse MAPK Phosphorylation Antibody Array kit (Raybiotech Inc), which was performed after 72 h of culture with or without neural CM treatment.^79^

### Osteogenic differentiation

Osteogenic differentiation medium consisted of neural CM or DMEM, 10% FBS, 1% penicillin/streptomycin with 100 nmol/L dexamethasone, 10 mmol/L β-glycerophosphate, and 50 μmol/L ascorbic acid (Sigma-Aldrich). Medium was changed every 3 days for 2 weeks. Alizarin red S (Sigma-Aldrich) staining was used to detect mineralization. Sodium hydroxide (0.1 N) was used to dissolve the calcium precipitate and quantified by absorbance at 548 nm.

### RNA isolation and quantitative real-time polymerase chain reaction

TRIzol (Life Technology, Waltham, Massachusetts) was used for total RNA isolation. Then, according to the manufacturer’s instructions, iScript cDNA Synthesis Kit (Bio-Rad, Hercules, California) was used to generate cDNA from RNA. SYBR Green PCR Master Mix (Life Technology) was used for quantitative real-time polymerase chain reaction (qRT-PCR). Primer information is provided in Table S2. *n* = 3 wells per group, and all studies were performed in three biological replicates.

### Single cell RNA sequencing (scRNA-Seq)

Four left femurs and tibias were dissected from ND and HFD groups at the experimental endpoint for scRNA-Seq. The bones were digested in a pre-warmed dissociation solution [3 mg/mL collagenase I (Worthington) and 4 mg/mL dispase II (Roche) in PBS] for 1.5 h at 180 r/min at 37 °C, run through a 40 μm filter (VWR) and rinsed with 3 mL medium [αMEM (Gibco) with 20% FBS, 1% penicillin/streptomycin]. Periosteal cells were centrifuged at 300 × *g* for 30 min at 4 °C. The cell pellet was washed twice with PBS and spun for 10 min at 300 × *g* at 4 °C. Periosteal cells were then resuspended in HBSS at a concentration of ~1 000 cells/μL. Cell viability was assessed with Trypan blue exclusion on a Countess II (ThermoFisher Scientific) automated counter and showed >85% viability. Cells were sent to the JHMI Transcriptomics and Deep Sequencing Core. The library was generated using the 10X Genomics Chromium controller following the manufacturer’s protocol. Cell suspensions were loaded onto a Chromium Single-Cell A chip along with reverse transcription (RT) master mix and single-cell 3’ gel beads, aiming for 10 000 cells per channel. Following generation of single-cell gel bead-in-emulsions (GEMs), reverse transcription was performed, and the resulting Post GEM-RT product was cleaned up using DynaBeads^®^ MyOne^TM^ Silane beads. The cDNA was amplified, SPRIselect (Beckman Coulter, Brea, CA) cleaned and quantified then enzymatically fragmented, and size selected using SPRIselect beads to optimize the cDNA amplicon size prior to library construction. An additional round of double-sided SPRI bead cleanup was performed after end repair and A-tailing. Another single-sided cleanup was done after adapter ligation. Indexes were added during PCR amplification and a final double-sided SPRI cleanup was performed. Libraries were quantified by Kapa qPCR for Illumina Adapters (Roche) and size was determined by Agilent Bioanalyzer 2100. Read 1 primer, read 2 primer, P5, P7, and sample indices were incorporated per standard GEM generation and library construction via end repair, A-tailing, adapter ligation and PCR. Libraries were generated with unique sample indices (SI) for each sample. Libraries were sequenced on an Illumina NovaSeq 6000 SP 100 cycle (San Diego, CA). *CellRanger* was used to perform sample de-multiplexing, barcode processing, and single-cell gene counting (Alignment, Barcoding, and UMI Count) at the JHMI Transcriptomics and Deep Sequencing Core. Downstream analysis steps were performed using *Seurat Version 4.4.0*. Cells were first filtered to have >500 and <8 000 detected genes, as well as less than 20% mitochondrial transcripts. *SCTransform*, including regression for cell cycle scores derived using the *CellCycleScoring* function, and dimensional reductions using uniform manifold approximation and projection (UMAP) was performed using *Seura*t. Pathway activation or module scores were generated using the *AddModuleScore* function of *Seurat* using validated gene lists from KEGG pathways. Module scores for signaling pathways were performed using the Escape R package (v0.99.0). Gene sets were derived from the Hallmark library of the Molecular Signature Database and from previous publications.^76,100,101^

### Statistical analysis

Data are expressed as mean ± 1 SD. All statistical analyses were conducted using Prism (GraphPad). Comparisons between groups were analyzed by either a Student’s *t* test (unpaired) or ANOVA. Before performing ANOVA tests, the Shapiro–Wilk test was performed to check normality, and the Bartlett’s Test was used to check homogeneity of variances. The Tukey-Fisher LSD criterion was used to perform pairwise post-hoc testing. *P* values less than 0.05 were considered statistically significant.

## Supplementary information

Supplementary data

## Data Availability

All study data are included in the article and/or supplementary materials. Transcriptomic data that support the findings of this study have been deposited in Gene Expression Omnibus (GEO) under SuperSeries (GSE272612). DRG single cell sequencing data was obtained from GEO (GSE139088).
