# Supplementary material for: Reduced somatosensory innervation alters the skeletal transcriptome at a single cell level in a mouse model of type 2 diabetes
Source: Bone Res. 2025 Jul 4;13:67. doi: 10.1038/s41413-025-00436-x (PMC12227694; doi:10.1038/s41413-025-00436-x)
Supplement: Supplementary file 1 — Supplementary data [file 41413_2025_436_MOESM1_ESM.docx]

**Supplementary data**


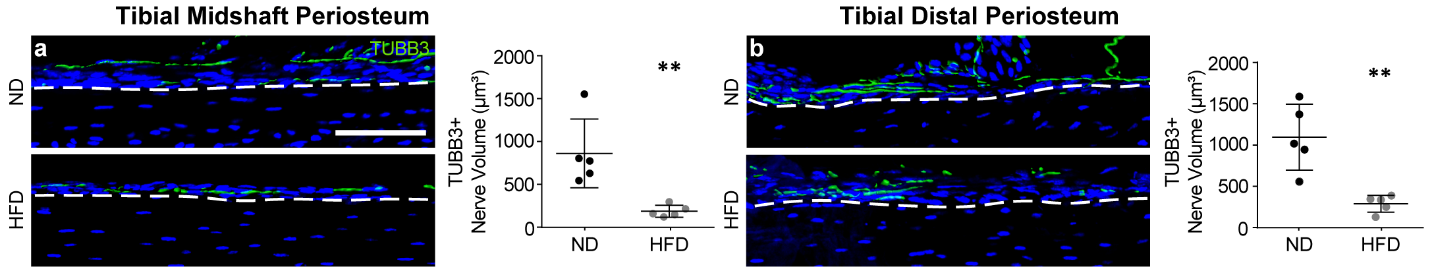


**Fig. S1. High-fat diet (HFD) feeding induces periosteal bone neuropathy.**

Normal diet (ND) or HFD feeding in C57BL/6J mice was instituted on week 4 of life, with analysis at week 16 of life. (**a**) The innervation of the tibial midshaft periosteum is visualized through the pan-neural marker β III-tubulin (TUBB3) immunostaining, represented in green. (**b**) The innervation of the proximal tibial periosteum is visualized through the pan-neural marker β III-tubulin (TUBB3) immunostaining, represented in green. n = 5. Scale bar: 100 µm. Graphs represent average values ± 1 SD, ***P*< 0.01. Comparisons between groups were analyzed by unpaired Student’s t test.


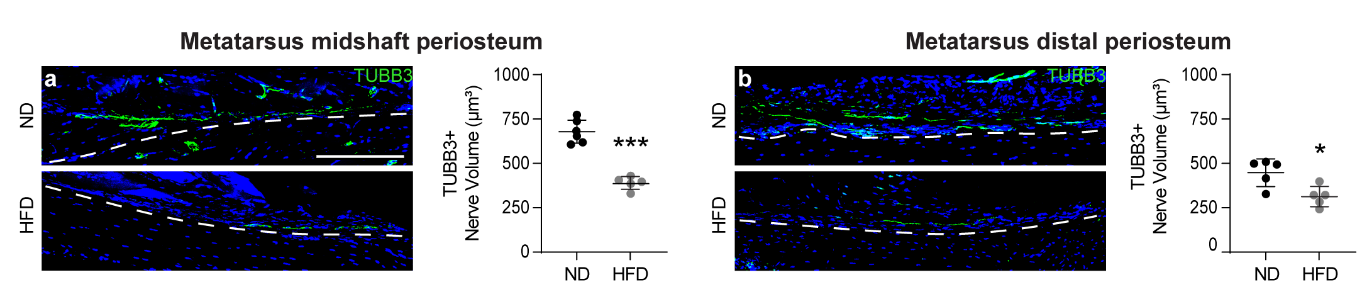


**Fig. S2. HFD feeding induces neuropathic change in the metatarsal periosteum.**

(**a**) Immunostaining and quantification of innervation of the metatarsal midshaft periosteum as visualized through the pan-neural marker β III-tubulin (TUBB3) immunostaining, represented in green. (**b**) TUBB3 immunostaining and quantification of innervation of the distal metatarsal periosteum. n = 5. Scale bar: 100 µm. Graphs represent average values ± 1 SD, **P*<0.05 and ****P*<0.001. Comparisons between groups were analyzed by unpaired Student’s t test.


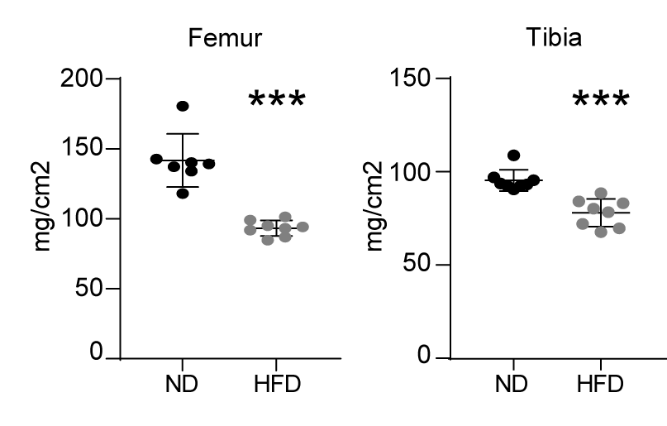


**Fig. S3. HFD feeding reduces bone mineral density.**

Quantification of DXA imaging in femur and tibia among ND and HFD-fed animals after 12 wks of dietary treatment. n =7 and 8 for ND and HFD-fed mice, respectively. Graphs represent average values ± 1 SD, ****P*<0.001. Comparisons between groups were analyzed by unpaired Student’s t test.


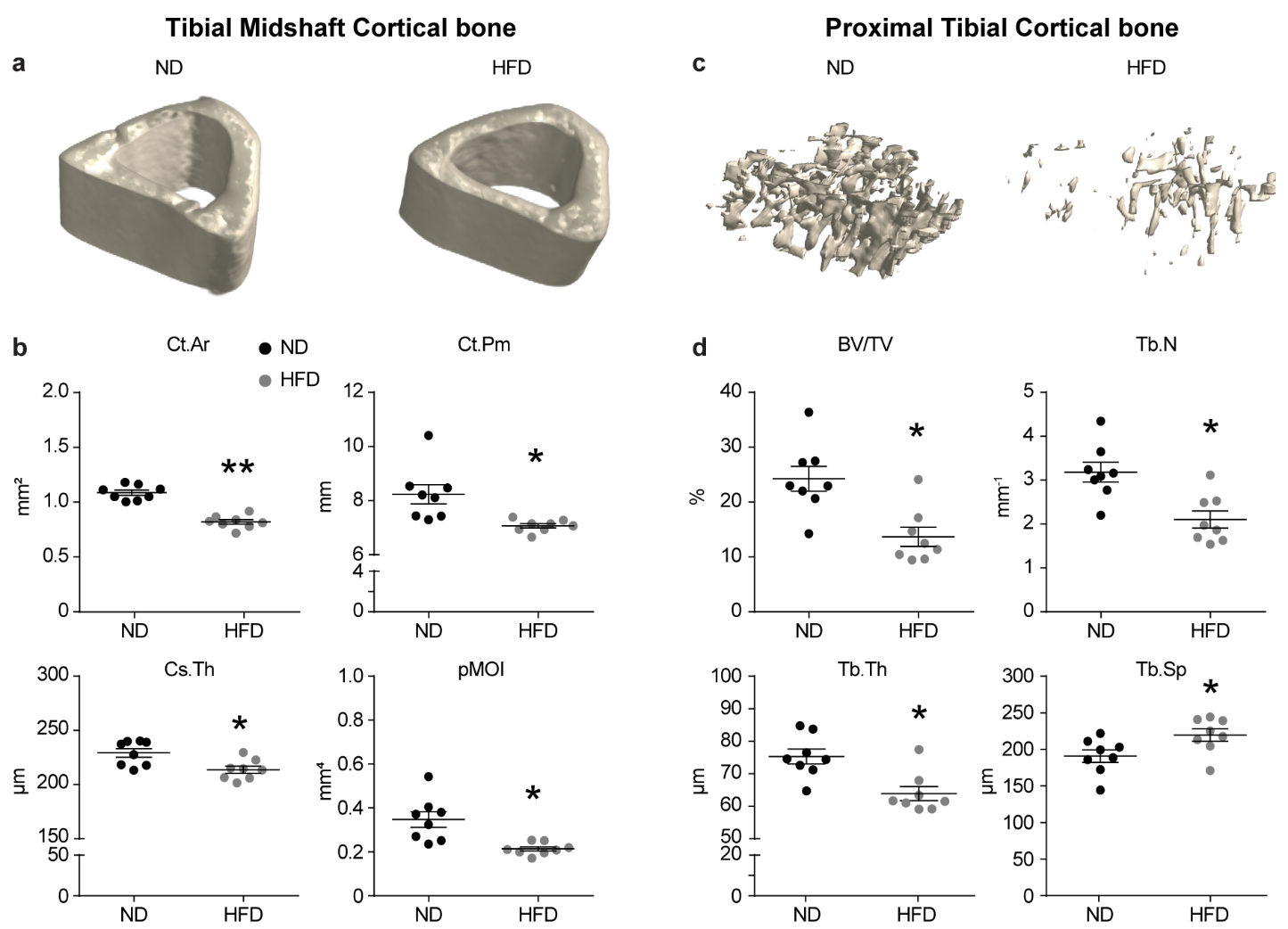


**Fig. S4. HFD feeding alters bone structural parameters of the tibia.**

(**a**) µCT images of tibial midshaft cortical bone following 12 wks of dietary treatment. (**b**) µCT quantifications of cortical area (Ct.Ar), cortical perimeter (Ct.Pm), cross-sectional thickness (Cs.Th) and polar moment of inertia (pMOI). n = 8 mice / group. (**c**) µCT images of distal tibial trabecular bone following 12 wks of dietary treatment. (**d**) µCT quantifications of Bone volume per total volume (BV/TV), Trabecular thickness (Tb.Th), Trabecular number (Tb.N) and trabecular separation (Tb.Sp) among ND and HFD-fed groups. n = 8 mice / group. Graphs represent average values ± 1 SD, **P*<0.05, ***P*<0.01. Comparisons between groups were analyzed by unpaired Student’s t test.


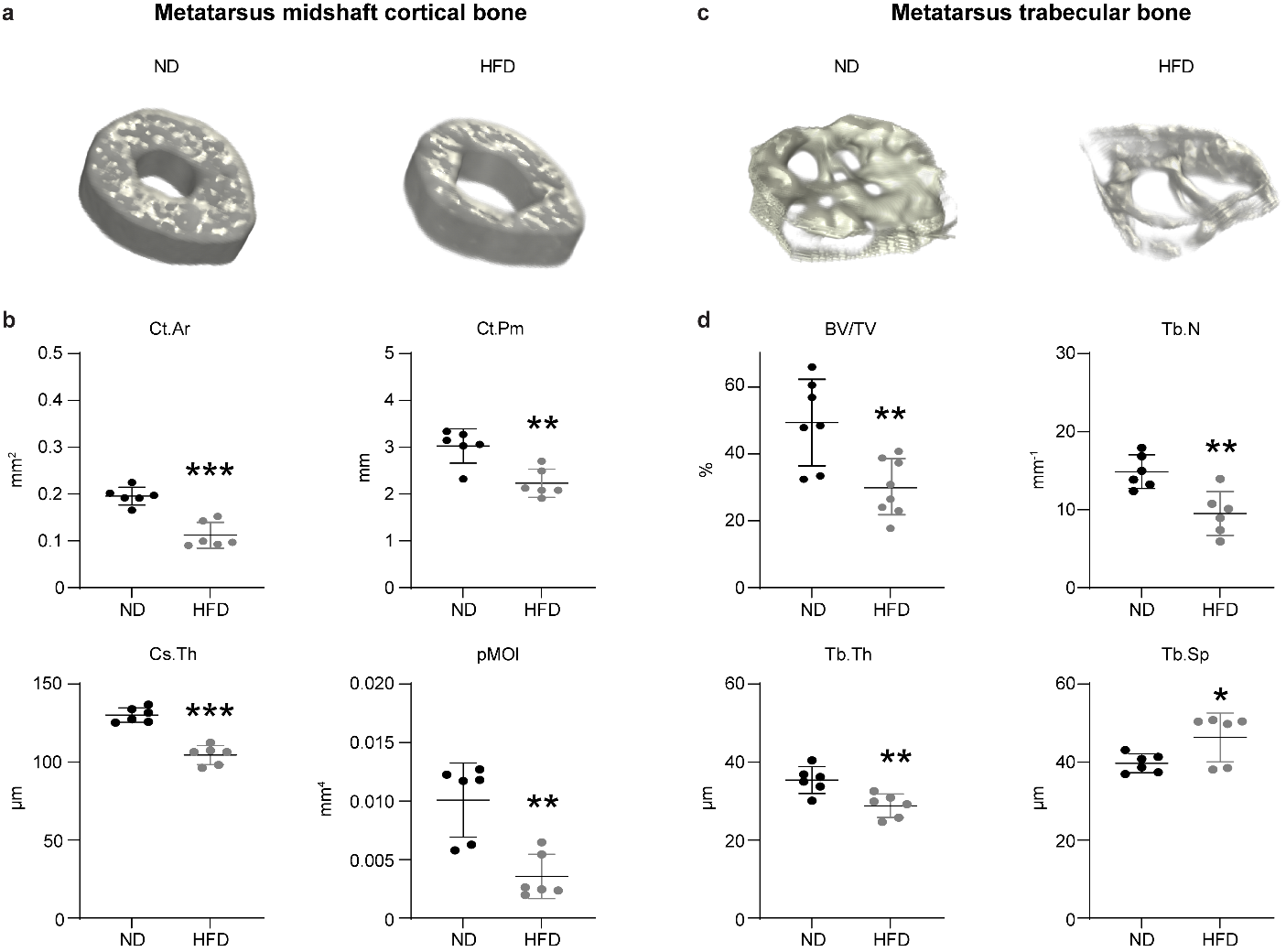


**Fig. S5. HFD feeding alters bone structural parameters of the 1st metatarsus.**

(**a**) µCT images of 1^st^ metatarsal midshaft cortical bone following 12 wks of dietary treatment. (**b**) µCT quantifications of cortical area (Ct.Ar), cortical perimeter (Ct.Pm), cross-sectional thickness (Cs.Th) and polar moment of inertia (pMOI). n=6 mice / group. (**c**) µCT images of distal 1^st^ metatarsal trabecular bone following 12 wks of dietary treatment. (**d**) µCT quantifications of Bone volume per total volume (BV/TV), Trabecular thickness (Tb.Th), Trabecular number (Tb.N) and trabecular separation (Tb.Sp) among ND and HFD groups. n = 6 mice / group. Graphs represent average values ± 1 SD, **P*<0.05, ***P*<0.01 and ****P*<0.001. Comparisons between groups were analyzed by unpaired Student’s t test. ND: Normal diet; HFD: High-fat diet.


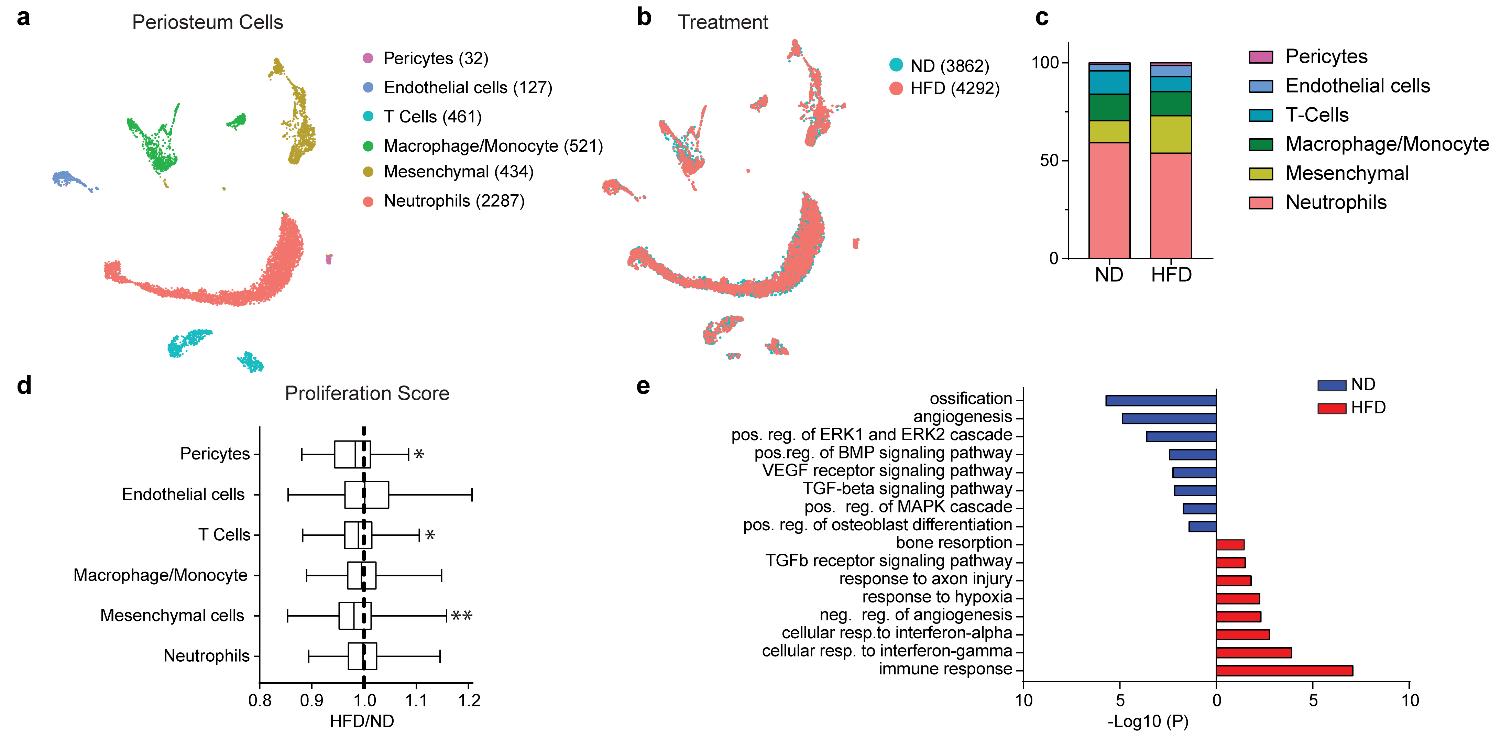


**Fig. S6. High-fat diet (HFD) feeding disturbs the skeletal cell phenotype.**

12 weeks after the initiation of the dietary treatment, four left femurs and tibias from ND and HFD mice were dissected. Periosteal cells were isolated, and scRNA-sequencing and analysis were performed. (**a**) UMAP visualization femoral and tibial periosteal cells clusters and (**b**) by treatment with HFD or ND feeding. (**c**) Cellular composition of cell clusters based on dietary treatment. (**d**) Analysis of proliferation module score across periosteum cell clusters under ND or HFD feeding conditions. Values greater than 1 indicate increased signaling activation in HFD group while values less than 1 indicate reduced expression among the HFD group. (**e**) Gene ontology (GO) term enrichment of the total periosteal cellular niche based on dietary treatment condition. Blue indicates upregulated GO terms with ND. Red indicates upregulated GO terms with HFD.


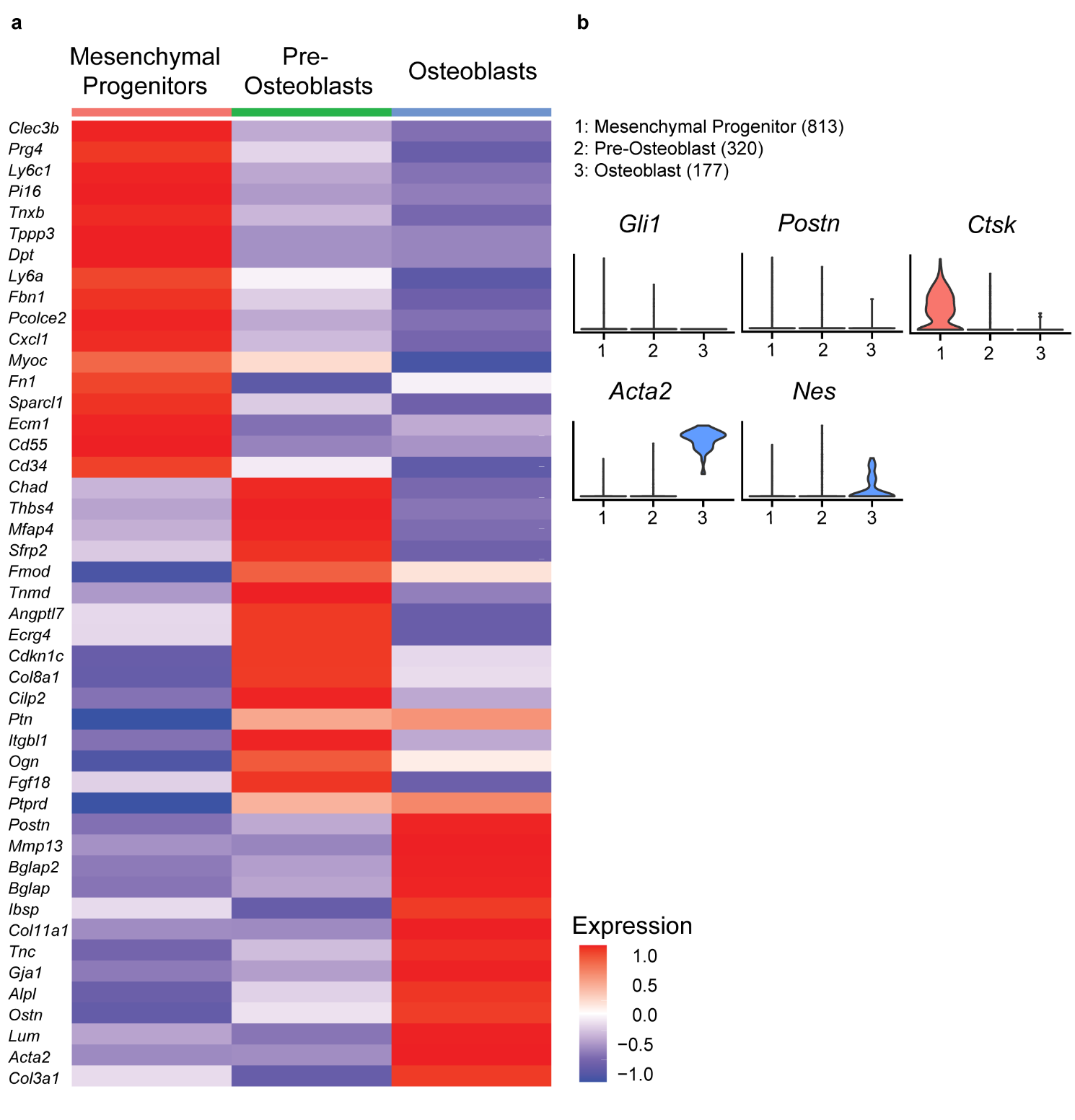


**Fig. S7. Mesenchymal cell subclusters DEGs.**

(**a**) Heatmap displaying top 14 DEGs within mesenchymal progenitors, pre-osteoblasts and osteoblasts clusters. (**b**) Violin plots showing the expression of characteristic periosteum cell markers within mesenchymal progenitors, pre-osteoblasts and osteoblast subclusters.


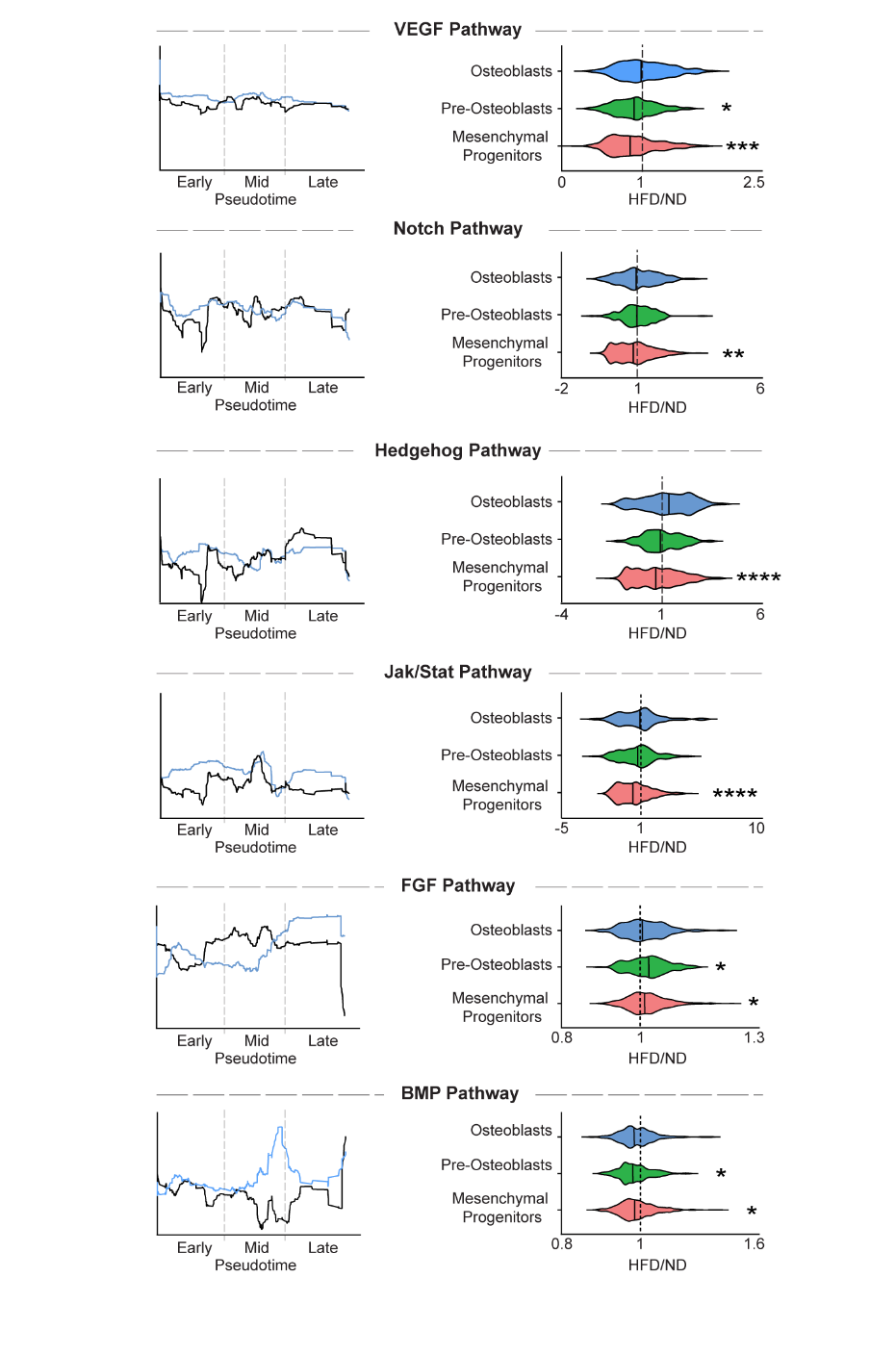


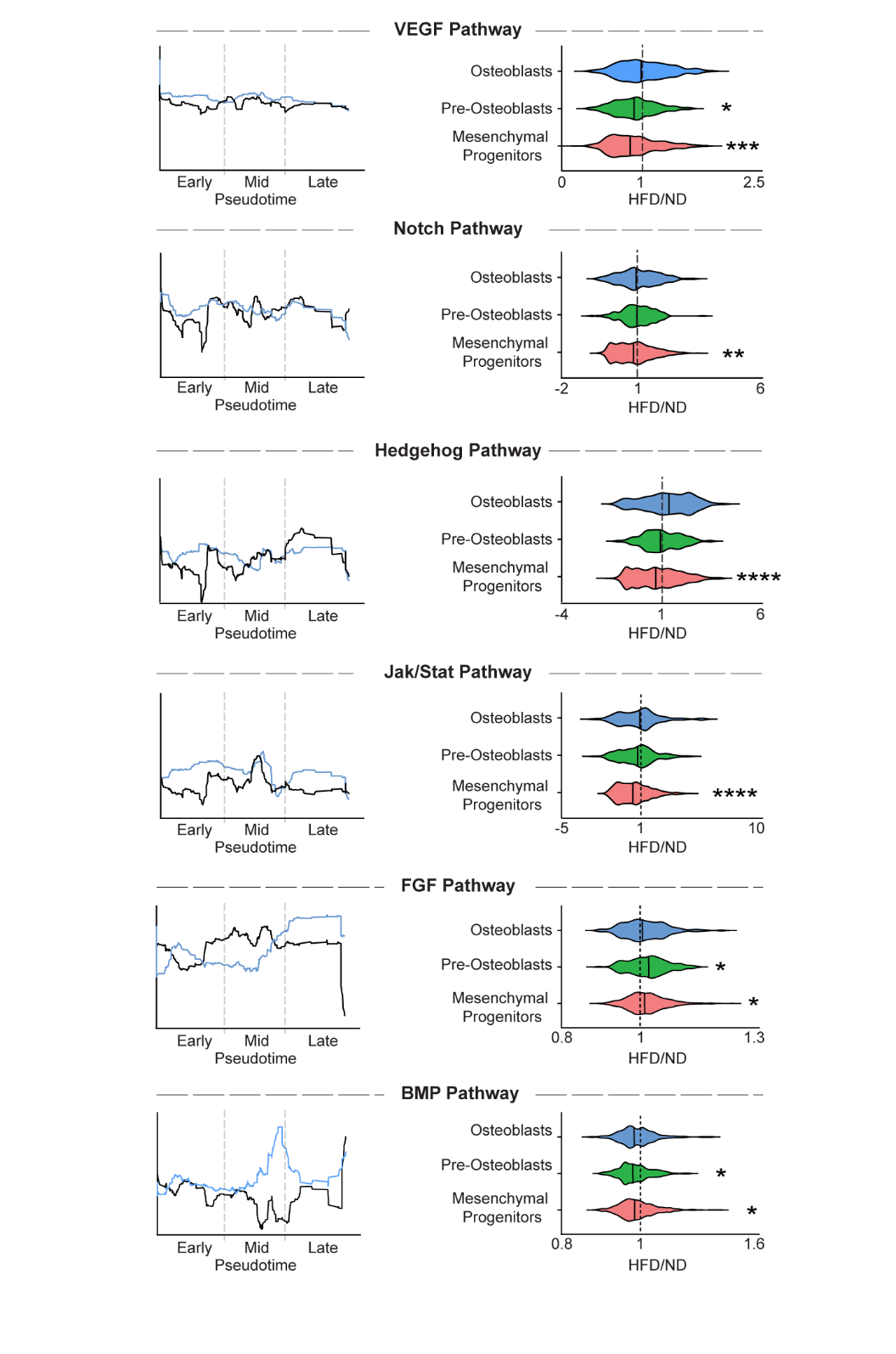


**Fig. S8. Additional pathways analysis.**

Linear graph analysis of dysregulated signaling pathways, including VEGF, Hedgehog, Notch, Jak/Stat, FGF and BMP signaling across pseudotime among ND (blue line) and HFD (black line) fed groups and module index scoring of mesenchymal cell subclusters. Dashed grey lines in module score graphs represent early, mid, and late pseudotime. Graphs represent average values ± 1 SD. Module scoring data was analyzed using the Kolmogorov-Smirnov test. **P*<0.05, ***P*<0.01, ****P*<0.001 and *****P*<0.0001 in comparison to ND control. 1,255 total mesenchymal cells analyzed.


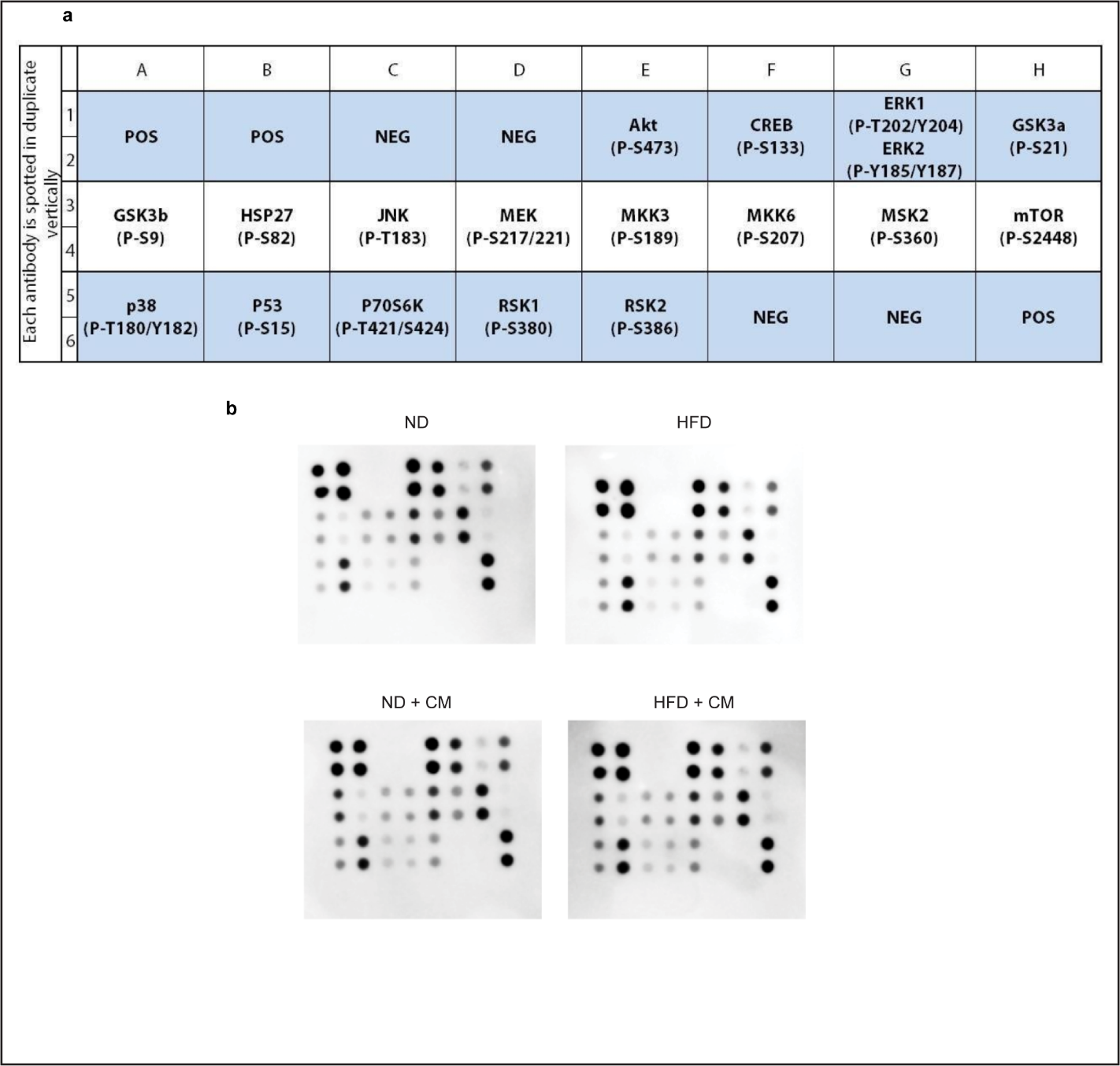
**Fig.** S9. MAPK phospho-protein array**.**

After 72 hrs of culture with or without neural CM treatment a MAPK phospho-protein array among ND and HFD derived periosteal cells. (**a**) MAPK signaling membrane map. (**b**) Developed chemiluminescent membranes for ND, HFD, ND+CM and HFD+CM conditions.

**Table S1. Antibodies used for immunohistochemistry.**

| **Antibody name** | **Manufacturer** | **Catalog #** | **Dilution ratio** |
| --- | --- | --- | --- |
| Anti-Tubulin beta 3 class III (TUBB3) | Abcam | AB18207 | 1:1,000 |
| Anti-Calcitonin Gene Related Peptide antibody (CGRP) | Sigma | C8198 | 1:200 |
| Anti-Tyrosine Hydroxylase Antibody (TH) | Sigma | AB152 | 1:1,000 |
| Anti- Protein gene product (PGP 9.5) | Abcam | ab108986 | 1:1,000 |

**Table S2. Quantitative PCR primers used.**

| **Gene** | **Forward** | **Reverse** |
| --- | --- | --- |
| *GAPDH* | 5’-CTGGGCTACACTGAGCACC-3’ | 5’-AAGTGGTCGTTGAGGGCAATG-3’ |
| *RUNX2* | 5’-TGGTTACTGTCATGGCGGGTA-3’ | 5’-TCTCAGATCGTTGAACCTTGCTA-3’ |
| *ALPL* | 5’-CCAGAAAGACACCTTGACTGTGG-3’ | 5’-TCTTGTCCGTGTCGCTCACCAT-3’ |

|  | **Ct.Ar (mm^2^)** | | | **Ct.Pm (mm)** | | | | **Ct.Th (µm)** | | | **pMOI (mm^4^)** | | |
| --- | --- | --- | --- | --- | --- | --- | --- | --- | --- | --- | --- | --- | --- |
|  | Mean (SD) | | **% Δ** | Mean (SD) | | **% Δ** | Mean (SD) | | | **% Δ** | Mean (SD) | | **% Δ** |
|  | ND | HFD |  | ND | HFD |  | ND | | HFD |  | ND | HFD |  |
| **Femur** | 1.08 (0.06) | 0.95 (0.05) | **-11.96** | 10.04 (0.37) | 9.64 (0.33) | **-4.01** | 216.46 (13.25) | | 198.3 (6.98) | **-8.38** | 0.58 (0.06) | 0.49 (0.06) | **-16.32** |
| **Tibia** | 0.94 (0.09) | 0.75 (0.05) | **-19.27** | 8.23 (1.00) | 7.07 (0.22) | **-14.15** | 229.22 (11.41) | | 213.69 (9.21) | **-6.77** | 0.34 (0.10) | 0.21 (0.02) | **-38.60** |
| **Metatarsus** | 0.19 (0.01) | 0.11 (0.02) | **-42.54** | 3.02 (0.36) | 2.23 (0.29) | **-26.49** | 130.19 (4.67) | | 104.55 (6.10) | **-19.68** | 0.01 (0.003) | 0.003 (0.002) | **-64.77** |

**Table S3. Change of cortical bone architecture in femur, tibia, and metatarsus.** n = 6-8 per group. Absolute mean change was calculated by mean HFD value – mean ND value. Mean percentage change values were calculated by [(mean HFD value –mean ND value)/mean ND value] *100.

|  | **BV/TV (%)** | | | **Tb.Th (µm)** | | | **Tb.N  mm^-1^** | | | **Tb.Sp (µm)** | | |
| --- | --- | --- | --- | --- | --- | --- | --- | --- | --- | --- | --- | --- |
|  | Mean (SD) | | **% Δ** | Mean (SD) | | **% Δ** | Mean (SD) | | **% Δ** | Mean (SD) | | **% Δ** |
|  | ND | HFD |  | ND | HFD |  | ND | HFD |  | ND | HFD |  |
| **Femur** | 22.33 (5.44) | 12.80 (2.65) | **-42.67** | 74.52 (6.84) | 65.98 (5.47) | **-11.45** | 2.97 (0.54) | 1.92 (0.29) | **-35.11** | 198.43 (18.46) | 213.86 (8.13) | **7.77** |
| **Tibia** | 24.23 (6.4) | 13.67 (4.97) | **-43.58** | 75.32 (6.54) | 63.90 (6.13) | **-15.16** | 3.18 (0.62) | 2.10 (0.55) | **-33.93** | 190.37 (24.27) | 219.09 (24.25) | **15.08** |
| **Metatarsus** | 49.39 (12.94) | 29.87 (8.40) | **-39.52** | 35.40 (3.43) | 28.81 (3.03) | **-18.59** | 14.86 (2.15) | 9.51 (2.80) | **-35.98** | 39.69 (2.42) | 46.33 (6.23) | **16.72** |

**Table S4. Change of trabecular bone microarchitecture in femur, tibia and metatarsus.** n = 8 per group. Absolute mean change was calculated by mean HFD value – mean ND value. Percentage change values were calculated by [(mean HFD value –mean ND value)/mean ND value] *100.
